# Self-assembly of human latexin into amyloid-like oligomers

**DOI:** 10.1186/1472-6807-7-75

**Published:** 2007-11-08

**Authors:** Irantzu Pallarés, Clara Berenguer, Francesc X Avilés, Josep Vendrell, Salvador Ventura

**Affiliations:** 1 Departament de Bioquímica i Biologia Molecular, Facultat de Ciències, Institut de Biotecnologia i de Biomedicina, Universitat Autònoma de Barcelona, E-08193 Bellaterra, Spain

## Abstract

**Background:**

In conformational disorders, it is not evident which amyloid aggregates affect specific molecular mechanisms or cellular pathways, which cause disease because of their quantity and mechanical features and which states in aggregate formation are pathogenic. Due to the increasing consensus that prefibrillar oligomers play a major role in conformational diseases, there is a growing interest in understanding the characteristics of metastable polypeptide associations.

**Results:**

Here, we show that human latexin, a protein that shares the same fold with cystatin C, assembles into stable spherical amyloid-like oligomers that bind thioflavin-T and congo red similarly to common amyloid structures but do not evolve into fibrils. Latexin self-assembly correlates with the formation of a mostly denaturated state rather than with the population of partially structured intermediates during the unfolding process. The results suggest that unfolding of α-helix 3 might be involved in the transition of latexin toward amyloidotic species, supporting the notion of the protective role of the native protein structure against polymerization.

**Conclusion:**

Overall the data herein indicate that latexin could be a good model for the study of the structural and sequential determinants of oligomeric assemblies in protein aggregation processes.

## Background

Amyloidoses are a group of protein misfolding diseases characterized by the polymerization of normally innocuous and soluble proteins or peptides into insoluble proteinaceous deposits. No sequence or structural similarities are apparent between any of the proteins that display the ability to form amyloid aggregates in diseases like Alzheimer's disease, type II diabetes, systemic amyloidosis or the transmissible spongiform encephalopaties [1,2].

There is a striking difference between the amounts of amyloid depositions in various types of amyloid disorders. In systemic lysozyme amyloidosis, for example, the deposits can grow to kilogram quantities in the liver [3,4], whereas in neurodegenerative diseases, where the quantity of aggregates can be almost undetectable in some cases [5], there is no clear correlation between the amount of amyloid deposition and the clinical severity of the disease. In Alzheimer's patients, a significant cognitive impairment was observed in the absence of noticeable amyloid deposits in the brain, although the levels of soluble amyloid oligomeric assemblies was found to be greatly elevated [6,7]. Evidence is accumulating that prefibrillar aggregates are cytotoxic both *in vivo *and *in vitro *[7-9], although this question is still open to debate. It still is not evident, however, which particular amyloid structures induce cell death by specific molecular mechanisms and which play the role of "inert" material and cause disease due to their quantity or mechanical properties. In spite of extensive studies of the fibrillar state, mainly by solid state NMR and X-ray diffraction, scarce structural information is available, and there is an increasing interest in the understanding of the biochemical and biophysical properties of metastable polypeptide associations preceding the aggregated states of proteins. Here, we characterize the conformational stability of latexin, an endogenous vertebrate carboxypeptidase inhibitor, and show that it associates into metastable oligomers with amyloidotic properties. Human latexin consists of two topologically equivalent subdomains, each one with a cystatin-like topology, consisting of an α-helix enveloped by a curved β-sheet. These subdomains are packed against each other through the helices and linked by a connecting segment encompassing a third α-helix [10,11]. Latexin adds up to other proteins unrelated to any known human disease able to form assemblies with the characteristics of amyloid [12]. Latexin oligomerization does not depend on the population of partially structured conformations but probably on the transition of a native helical structured region to an unfolded conformation under conditions that are selectively unfavorable for the folding and nativeness of this polypeptide. In this particular case, conditions promoting self-assembly into highly stable oligomers do not promote further evolution into fibrillar structures suggesting that, in protein deposition, the intermolecular contacts responsible for the final, usually highly ordered, structure of protein aggregates could not necessarily coincide with those promoting the formation of the initial oligomeric assemblies, which may have important consequences for the therapeutics of amyloid diseases.

## Results

### Conformational stability of latexin

**Figure 1:**
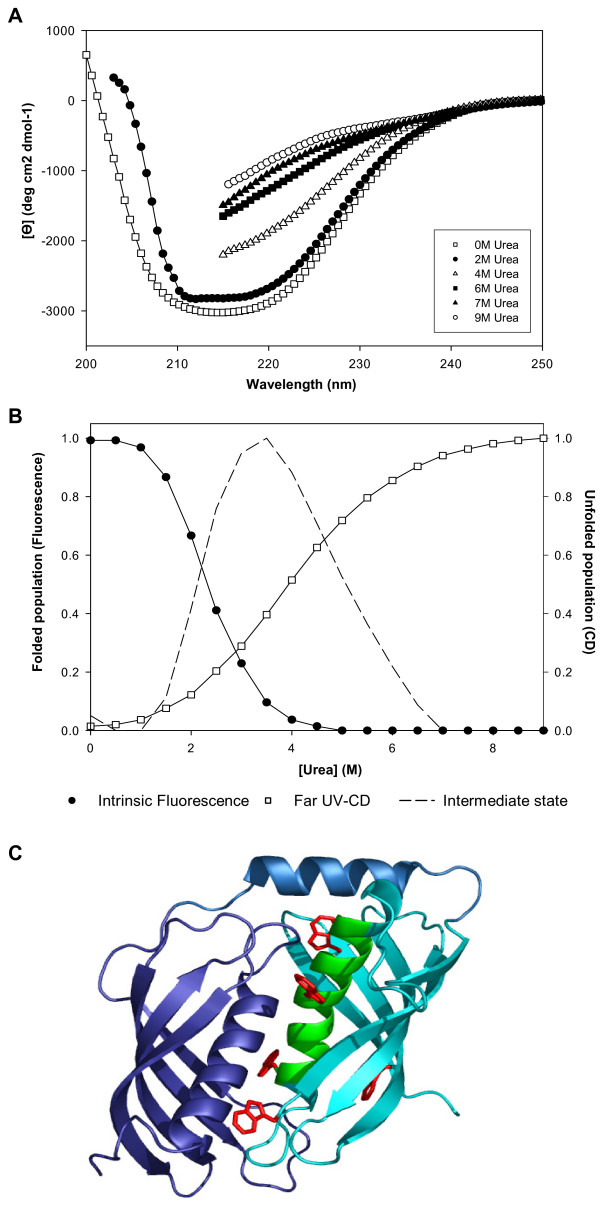
**Conformational properties of latexin**. A) CD spectrum of latexin at increasing urea concentrations. B) Overlay of the urea denaturation curve monitored by intrinsic fluorescence and by far UV-CD. The difference in the two transition midpoints is usually associated with the presence of an intermediate state whose population is theoretically calculated from the normalized unfolding curves. C) 3D structure of latexin (2BO9). The five triptophans (red) are located in the C-terminal subdomain (light blue and green) and the region corresponding to the main hot spot is located in the alpha-helix 3 (green).

The far-UV circular dichroism (CD) spectrum of latexin recorded under native conditions is consistent with the recently determined 3D crystal structure, which corresponds to a well-structured protein with an α/β topology [11]. According to the CD spectra this globular structure is progressively lost, however, in the presence of increasing concentrations of urea (Figure 1A). The dependence of secondary structure on urea concentration, measured as the mean residue ellipticity at 220 nm, is shown if Figure 1B. The equilibrium denaturation curve presents a single sigmoidal transition corresponding to a two-state unfolding reaction with a midpoint at 4.1 M urea. This contrasts with a much lower transition midpoint (2.3 M urea) deduced from the sigmoidal curve representing the urea-induced changes in the intrinsic fluorescence of tryptophan residues in latexin, an alternative and even better reporter of tertiary structural changes. The difference in the two transition midpoints is usually associated with the presence of an intermediate state whose population can be calculated from the normalized unfolding curves (Figure 1B, broken line). The intermediate state forms between 1.5 and 6.5 M urea concentration with a maximum population at 3.5 M. At this urea concentration the intermediate keeps most of the secondary structure of the native latexin. It is worth mentioning that latexin exhibits a striking asymmetry of tryptophan distribution between its two subdomains. All five tryptophan residues are located in the second subdomain of latexin, four of them shielded at the interface between both subdomains, the remaining tryptophan being exposed to the solvent and presumably contributing little to variations in fluorescence upon unfolding (Figure 1C). Therefore, changes in fluorescence emission may only reflect local conformational rearrangements, such as a loss of contacts between subdomains, rather than a global unfolding of the protein or of one of its subdomains. This is also likely from the study of binding of latexin to TNS (2-p-toluidinylnaphthalene-6-sulfonate), a dye used to probe the exposure in partially folded states of previously hidden hydrophobic clusters. TNS emission spectra were recorded as a function of urea concentration and no significant binding could be detected in the folded (0 M urea) and completely unfolded (9 M urea) states or in the range 3–4 M urea where the intermediate maximally populates (data not shown).

### Latexin self-association

**Figure 2:**
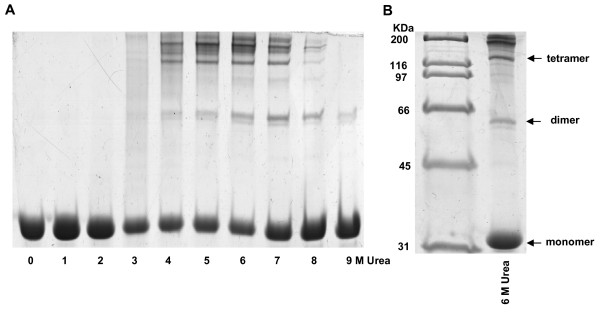
**SDS-PAGE electrophoresis of latexin in various concentrations of urea**. A) Isolated recombinant latexin was incubated at 1 mg/ml for 24 hours at room temperature at increasing urea concentrations. The incubation mixtures were analyzed by electrophoresis in 12% SDS-PAGE. B) Latexin incubated at 6 M urea for 24 hours at room temperature and after the urea was removed and incubated for 12 hours.

In a number of cases, in particular for cystatins [13], the appearance of folding intermediates has been linked to the self-association of polypeptides into oligomers that further evolve to yield insoluble amyloid-like structures. To asses whether the accumulation of an intermediate during the unfolding of latexin could result in the formation of stable oligomeric species and to simultaneously detect the formation of oligomers of different sizes, samples of latexin incubated for 24 h at different urea concentrations were analyzed by SDS-PAGE electrophoresis. The results shown in Figure 2 demonstrate that latexin self-associates into SDS-resistant oligomers (dimers, tetramers and higher molecular weight species) in an urea-dependent manner. SDS-resistant self-associated latexin oligomers start to appear at 3–4 M urea and accumulate maximally at 6–7 M urea. A decrease in oligomer population is observed at higher urea concentrations. 9 M urea results in strong denaturing conditions where only very-tight interactions can be maintained. In this denaturant concentration the oligomeric forms are mostly constituted by dimeric species, whereas larger associations observed at lower urea concentrations, are mostly absent, suggesting that the higher order oligomers are formed by multiple of two polypeptide chains and, that the contacts within a dimmer are stronger than between dimers. Interestingly enough, during amyloidogenesis of the human cystatin stefin B, dimers have been shown to be the initially formed species and subsequent oligomerization could involve the direct association of these bulding blocks into tetrameric and higher order species in which dimers become entwined [14]. The two cysteine residues in latexin are not involved in a disulfide bond in the native state and neither are responsible for the association of latexin in the presence of urea since the distribution of oligomers does not change in the presence of 2-mercaptoethanol. The distribution of the oligomeric species in samples incubated in the presence of urea, does not change significantly upon removal of the denaturant indicating that the aggregation is not reversible. From the comparison of the polymerization and unfolding data it can be inferred that, in the particular case of latexin, oligomerization at low urea concentrations is unfavorable because protein folding competes with and suppresses self assembly whereas the significant decrease in the amount of oligomers at urea concentrations above 7 M indicates that high chaotropic agent concentrations are unfavorable to both the native and self-assembled states of latexin.

**Figure 3:**
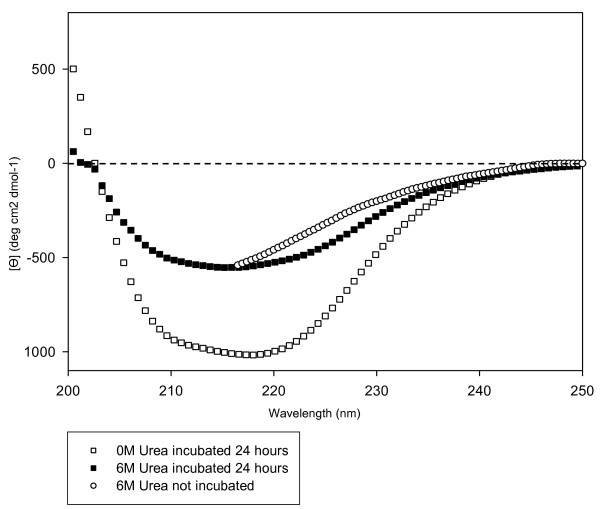
**Latexin secondary structural changes after 24 hour incubation in urea monitored by CD**. Far-UV circular dichroism spectra were measured at 25°C for samples at 0 M urea incubated 24 hours; 6 M urea incubated 24 hours; 6 M urea not incubated.

To understand the conformational changes occurring during oligomerization, samples incubated for 24 hours at 0 M and 6 M urea were desalted and their far-UV circular dichroism spectra compared with that of fresh samples containing the same amount of urea (Figure 3). The CD spectrum of the incubated and fresh samples are indistinguishable in the absence of urea. The CD spectra of the 6 M urea incubated sample is typical of a largely extended β-sheet conformation as revealed by the single negative band at 217–220 nm. This spectrum differs in shape from the native conformation of latexin, which is also rich in β-sheet structure. According to the estimation of protein secondary structure content using the Contin method with the CDPro suite [15], 6 M urea incubated latexin is devoid of any α-helical secondary structure. Accordingly, this sample after urea removal does not exhibit any inhibitory activity against carboxypeptidase A (data not shown), indicating the absence of molecules in native conformation in the protein solution and confirming the irreversibility of the aggregation process. The fresh 6 M urea sample displays a CD spectrum corresponding to the much more unfolded conformation already commented upon in Figure 1A. This suggests that the self-association of latexin probably begins with the establishment of intermolecular interactions between primary unfolded molecular segments in the monomeric species that lead to the subsequent build up of a predominant, highly stable, β-sheet conformation.

### Amyloid-like features of latexin oligomers

**Figure 4:**
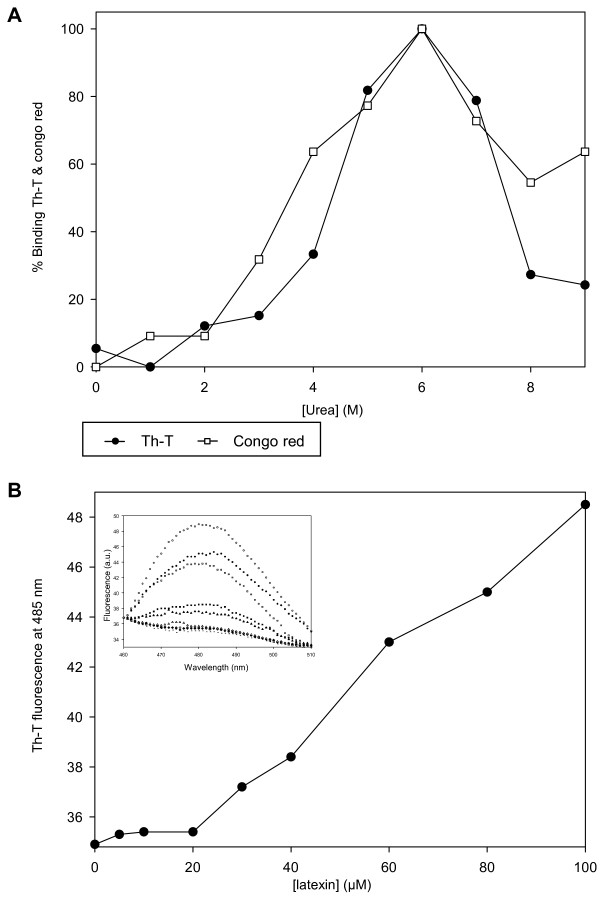
**Binding of amyloid dyes by latexin**. A) Changes in the fluorescence emission of Th-T and in congo red absorbance upon binding to latexin (0.1 mg/ml) after 24 hours incubations at various concentrations of urea and at 25°C. B) Th-T fluorescence at 485 nm at various latexin concentrations. (Inset) Fluorescence emission spectra of th-T at various latexin concentrations.

The stability and the extended β-sheet conformation of latexin self-assemblies suggested that they might have amyloid-like properties. Binding of thioflavin-T (Th-T) to amyloid aggregates induces a large increase in the fluorescence of Th-T relative to the free dye [16]. In a binding assay of Th-T to latexin previously incubated for 24 hours in the presence of different urea concentrations, changes in the fluorescence intensity of the dye at 485 nm are only detected above 2 M urea, with a maximum relative increase at 6 M urea and a progressive decrease at higher urea concentrations (Figure 4A). Incubation of latexin at concentrations above 20 μM suffices to promote the formation of Th-T positive species after one day in 6 M urea (Figure 4B). This agrees with the proposal of the existence of a critical concentration for amyloid assembly, which is specific for each particular system [17], since a linear correlation exists between the formation of Th-T binding forms and the initial amount of protein in the solution above the 20 μM threshold.

Congo red is another amyloid dye, with an absorbance maximum at 490 nm, that increases and shifts to red upon binding to amyloid material. We assayed congo red binding to latexin after protein incubation for one day at different chaotropic agent concentrations. In a fashion very similar to what is observed with Th-T, maximum binding occurs at 6 M urea whereas little binding is detected in protein solutions below 2 M urea and the binding decreases in solutions above 6 M urea (Figure 4A). Latexin incubated at 6 M urea promoted a maximum change in the congo red spectra at 533 nm, similar to that observed for aged solutions of Aβ amyloid peptide [18]. The binding of latexin solutions to both amyloidotic dyes closely correlates with the amount of oligomeric forms they contain, as analyzed by SDS-PAGE. In agreement with the CD data, this indicates that latexin oligomers display amyloid-like conformations. Importantly, the formation of these species correlates with the loss of secondary structure, but not with the formation of an intermediate or the population of native latexin. This observation is evident from the comparison of the traces in Figures 4A and 1B.

**Figure 5:**
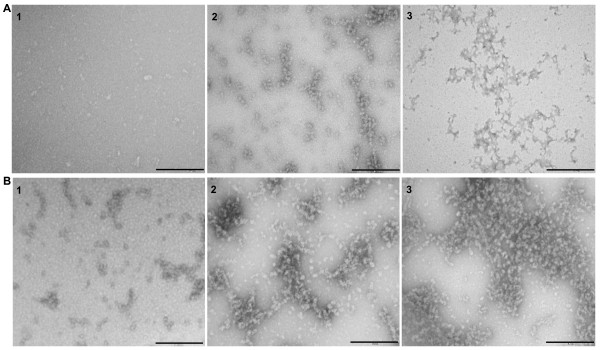
**Representative images of latexin protein incubated at various urea concentrations for more than one day**. A) Representative EM images of latexin samples incubated during: (1) 24 hours at 0 M urea; (2) 24 hours at 6 M urea; (3) 24 hours at 9 M urea. B) EM images of latexin samples incubated at 6 M urea during: (1) 24 hours; (2) one week; (3) one month. The scale bar represents 500 nm.

The morphologies of the latexin species formed under different experimental conditions were examined by transmission electron microscopy (EM) (Figure 5). In agreement with the above mentioned results, little deposition or particles were detected for latexin incubated 24 h in solutions below 2 M urea. Latexin incubated in 6 M urea exhibited numerous roughly spherical aggregates with average diameter of 18 nm (with larger particles reaching diameters up to 45 nm) whereas samples incubated in 9 M urea mainly display amorphous aggregates. To determine if the morphology of oligomers formed at 6 M urea evolve with time, samples of latexin incubated for one week and one month were imaged using EM. As can be seen in Figure 5B, the round-shaped oligomers tend to grow in size and co-associate in chains and networks after one week and the observation is even more clear after one month incubation. No evolution to fibrillar structures was observed in the preparation.

### A "hot spot" of aggregation in α-helix 3 of latexin

**Figure 6:**
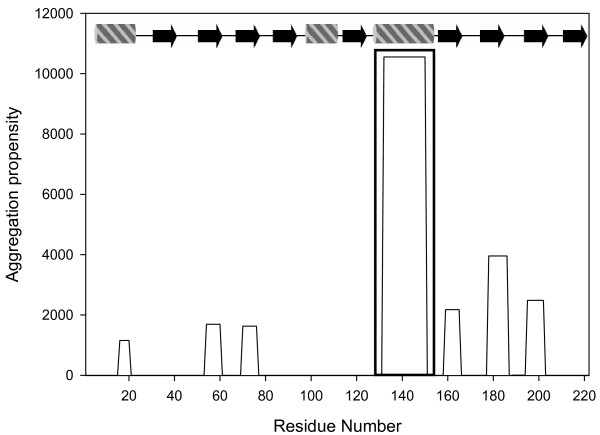
**Aggregation profile of human latexin**. The aggregation profile was generated using the program AGGRESCAN. The region that displays the highest aggregation propensity, T134-W149, is framed. The regular secondary structure elements shown, correspond to the human latexin structure, cylinders stand for α-helices and arrows for β-strands.

**Figure 7:**
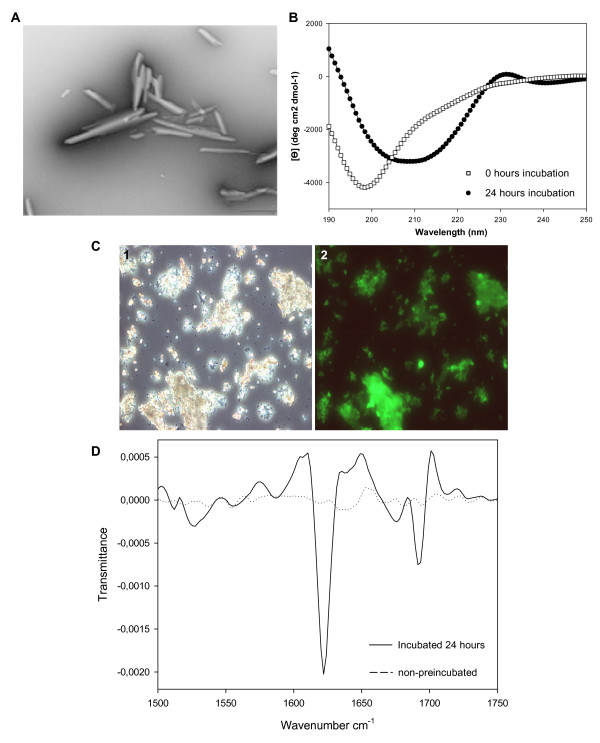
**Analysis of VLHLAWVA peptide self-assembly**. A) An electron micrograph of the VLHLAWVA microcristals. The scale bar represents 1 μm. B) CD spectrum of VLHLAWVA after 0 hours of incubation and 24 hours at 25°C in buffer P. C) Thioflavin-T fluorescence of stained material after one day incubation in buffer P. (1) Phase contrast microscopy. (2) Fluorescence microscopy under UV-light. D) FTIR spectrum of the non-preincubated peptide in buffer P and the peptide incubated in buffer P 24 hours. The positions of the spectral components are estimated from the second derivate analysis.

Polypeptide sequences appear to contain local regions that are "sensitive" for aggregation. It is possible to describe with considerable accuracy the *in vitro *amyloid aggregation propensities of polypeptides using algorithms that take into account the physico-chemical properties of their sequences [19-21]. This leads directly to the definition of "hot spot" of aggregation as a certain region that displays higher aggregation propensity than the rest of the sequence. Latexin forms oligomeric species from a mainly unfolded state since oligomerization occurs maximally at 6 M urea where according to the CD and intrinsic fluorescence analysis, latexin has lost its 3D structure and most of its secondary structure elements, being about 80% of the latexin population in unfolded conformations. Under these conditions aggregation-prone regions, if present, may promote and drive the self-association event. Using AGGRESCAN, an in-house developed algorithm that predicts sequence-dependent effects on the aggregation of proteins [22], we detected one main "hot spot" encompassing residues 127–156 (Figure 6) located in the α-helix 3 of latexin (see Figure 1C). The prediction is in excellent agreement with that obtained using TANGO algorithm [19]. Attempts to synthesize a peptide comprising residues T134-W149 within the "hot spot" region in order to confirm its hypothetical critical role in aggregation failed because of its high intrinsic aggregation tendency. However, a shorter 8-residue peptide corresponding to the central part of the "hot spot", V136-A143 (VLHLAWVA) was soluble enough in phosphate buffer. Over-night incubation of the 8-residue peptide at 500 μM in 5 mM sodium phosphate pH 7.5 yielded well-ordered microcrystals with average dimensions of 0.75 μm × 0.15 μm (Figure 7A). Binding of Th-T to VLHLAWVA microcrystals was tested using fluorescence microscopy. Areas rich in peptidic material appear stained with Th-T, giving a bright green-yellow fluorescence against a dark background suggesting an amyloid-like organization of the peptide in the crystals (Figure 7C). No fluorescence was detected for the buffer without sample. The structural analysis of VLHLAWVA microcrystals by Fourier transform IR shows a strong peak at 1620 cm^-1 ^dominating the amide I region spectrum. This feature, together with the presence of an additional sharp peak at 1690 cm^-1^, resulting from a splitting of the main β-sheet signal at 1620 cm-1 and usually used as diagnostic peak for the presence of antiparallel β-strands, allows to propose a highly ordered intermolecular β-sheet structure for the peptidic crystals (Figure 7D). Finally, far-UV CD analysis further indicates the transition of the peptide from a random conformation to a polymeric β-sheet structure upon incubation (Figure 7B). The presence of a new positive signal at 232 nm, usually attributed to aromatic contributions, in the incubated sample, strongly suggests that Trp6 is in a defined conformation and not randomly exposed to solvent in the assembled structure. Consequently, the data herein suggest that, although the sequence stretch containing the "hot spot" is located in a well folded and highly packed helical structure in the native protein, it might also be involved in the transition of latexin from a monomeric globular conformation to amyloid-like oligomeric structures.

## Discussion

It has been proposed that almost every protein, when incubated under the right conditions, can aggregate [12]. Since globular proteins rarely aggregate from their native states, their destabilization and subsequent increased population of unfolded molecules is well established as a triggering factor in disorders associated with the deposition of proteins that are globular in their normal functional states [23,24], like β2-microglobulin, lysozyme, transthyretin and the prion protein. Accordingly, many of the proteins involved in depositional disorders are mostly unstructured within the cell [24]. These include amylin, amyloid-β-protein, and α-synuclein, among others. In these cases, protein polymerization and deposition does not require unfolding and can occur by direct self-assembly of the unstructured polypeptide chains. In the last few years, proteins unrelated to any known human disease have been found to convert *in vitro *into higher order structures that also present a cross-β conformation and fulfill all characteristics of amyloid fibrils [25-27]. Latexin is a vertebrate carboxypeptidase inhibitor with an α/β fold, closely resembling that of cystatins. Interestingly enough, several members of the cystatin family have been shown to form stable oligomeric assemblies and amyloid fibrils [13,28,29].

The latexin fold is destabilized at low urea concentrations and the protein becomes mostly unfolded above 6 M urea. A stable equilibrium intermediate populates during the unfolding reaction of latexin. The intermediate most likely corresponds to an open form in which the two domains of latexin have lost their contacts but still conserve most of their secondary structure and probably domain architecture. Nevertheless, this partially folded form of latexin does not appear to be competent for polypeptide self-assembly, since its formation does not overlap with conditions that promote maximum oligomerization. As previously described for myoglobin [30], the oligomerization of latexin correlates with conditions in which the protein is mostly unstructured, rather than with environments that generate partially folded intermediates. In the case of the structurally related cystatin C, the effect of temperature, pH and denaturant in self-assembly has been assayed [31]. In contrast to latexin, cystatin C self-assembles through the formation of a partially unfolded intermediate, under conditions quite far from those leading to the unfolding of the protein. The process of polymerization involves the initial formation of a dimer by domain-swapping [32] and subsequent propagation of this effect to form oligomeric assemblies [33]. Consistently, stabilization of the monomer by different means avoid swapping and further cystatin polymerization [34]. Although, in the case of latexin dimers are relevant forms in the oligomerization process, it is unlikely that they are domain-swapped forms, since these species essentially keep the same 3D protein structure as the monomer, while the assemblies of latexin have lost most of the native organization. Also, according to our data, oligomer formation by a mechanism in which dimers act as building blocks is more likely than a propagated (or run-away) mechanism. Overall, latexin differs from protein models in which polymerization depends on the population of partially structured conformations, usually enriched in β-sheet structure [27,35]. Instead, our data indicate that, like in the case of intrinsically unstructured proteins, the structural precursors in the first stages of latexin polymerization correspond to protein regions containing little, if any, secondary structure.

Provided that self-assembly takes place in conditions where the polypeptide chain is mainly unfolded, the use of prediction algorithms that forecast aggregation-prone regions merely from the protein sequence should allow the detection of the latexin region(s) involved in the formation and maintenance of intermolecular contacts. According to such programs, the latexin region with the highest probability to be involved in amyloid-like intermolecular contacts comprises residues T134-W149. This is supported by the observation that the central sequence of this predicted region, corresponding to the peptide VLHLAWVA, forms well-ordered microcrystals with amyloid properties as analyzed by EM, Th-T binding, CD and FTIR. The formation of peptide microcrystals in physiological conditions is usually associated with rapid formation of strong and specific intermolecular interactions. In the protein context the same strong contacts might become relevant for the oligomerization process of latexin. These microcrystals are of interest since their properties closely resemble those formed by peptides related to fibril-forming proteins like Alzheimer's amyloid-β, tau and prion proteins from which the high resolution atomic structure of a common amyloid cross-beta spine involving the formation of steric zippers has been determined [36,37]. Experiments are underway in our lab to solve the structure of this peptide assembly.

Overall, the data herein indicate that, in the case of latexin, the formation of oligomeric species occurs maximally under conditions that are specifically adverse to the folding of the protein. This can be easily rationalized, since aggregation-prone regions have their side chains usually hidden in the inner hydrophobic core of the native globular protein or already involved in the network of contacts that stabilizes the secondary and tertiary structure of polypeptides. Specifically, the VLHLAWVA region of latexin is located in α-helix 3, where the establishment of anomalous intermolecular interactions is blocked not only because it belongs to a secondary structure element, but also because its side chains participate in the interface contacts between the two domains of latexin. From the data shown, it appears that opening of the interface is not sufficient to promote the loss of the α-helical structure and oligomerization, but is probably a prerequisite for this event to occur. Once in a non-structured context and exposed to solvent by denaturation, the VLHLAWVA region is ready for the establishment of intermolecular contacts as proven by the analysis of the eight-residue peptide. Thus, latexin constitutes yet another example that illustrates the protective role played by the protein native structure against aggregation.

## Conclusion

Protein aggregation generates a very broad range of structures including oligomers, protofibrils, and fibrils. Determining the role of these so-called "prefibrillar species" in assembly is of paramount importance, since such complexes have been proposed for many proteins to be responsible for the cellular toxicity associated with amyloid disease [38,39]. Dimeric, tetrameric and higher order latexin oligomers were visualized and characterized. From this study, it appears that the latexin system could be an excellent model for the isolation and deep structural and biochemical characterization of highly stable low-molecular weight oligomeric species that appear to be the final association state of latexin under strongly denaturing conditions, despite exhibiting tinctorial and conformational amyloid-like properties. The drastic conditions in which latexin self-assemblies only allow for strong intermolecular contacts to be established and probably prevent the formation of the specific network of non-covalent interactions needed to evolve into the quasi-crystalline structure characteristic of amyloid fibrils. Accordingly, urea has been used to reduce the driving force for Aβ-peptide aggregation, in an effort to isolate stable oligomeric assemblies [40]. Increasing urea concentrations reduced the average size of the aggregates, and the morphology of the aggregates changed from linear fibrils to globular oligomeric structures This behavior indicates that the interactions involved in the formation of the primary, low molecular weight oligomers during protein aggregation do not necessarily overlap with those driving the formation and stabilization of higher order fibrillar assemblies. This is relevant for the therapies of amyloid disease, since it suggests that drugs designed to interfere with the interactions occurring in amyloid fibrils would not necessarily have a significant effect in the initial assembly of pre-fibrillar aggregates, which have been implicated as the toxic species in conformational disorders.

## Methods

### Materials

Recombinant latexin was produced in *E. coli *and purified as previously described [11]. The peptide VLHLAWVA was synthesized by the American Peptide Company, Sunnyvale, CA. Previously a peptide comprising T134-W149 was tried to be synthesized by the same company but its high aggregation tendency made it impossible to obtain it in a soluble form for further purification. Urea, thioflavin-T and congo red were purchased from Sigma. Unless otherwise mentioned, all solutions used for latexin analysis were made in 50 mM phosphate buffer pH 7.5 (buffer N), and 5 mM phosphate buffer pH 7.5 was used for peptide preparations (buffer P). All the experiments were performed at 25°C.

### Sample preparation

Unless stated otherwise, oligomeric samples were obtained by incubating protein at 1 mg/ml in buffer N in the presence of different concentrations of urea from several hours to several days. The peptide VLHLAWVA was incubated at 500 μM concentration in buffer P for 24 h.

### Prediction of sensitive regions for aggregation

To determine which regions of latexin sequence could be involved in aggregation the in-house developed AGGRESCAN software was used [41] and the predictions cross-checked against TANGO [42] outputs. Both algorithms detected the sequence stretch around residues 130–150 as the main region which could be potentially involved in the aggregation of latexin from a mainly unfolded state [19,22]. A peptide covering residues V136-A143 (VLHLAWVA) was synthesized in order to check experimentally its relevance for latexin aggregation.

### Circular dichroism and fluorescence spectroscopies

Circular dichroism (CD) spectra in the far-UV region were obtained by using a Jasco-810 spectropolarimeter at 25°C. Spectra were recorded for native proteins and for proteins after incubation at different urea concentrations (ranging from 0 to 9 M) at 100 μg/ml protein concentration. Twenty accumulations were averaged to obtain each spectrum. The mean residue ellipticity at 220 nm was plotted versus urea concentration to obtain denaturation curves. The fitting of the experimental data was performed using the non-linear, least-squares algorithm provided with the software KaleidaGraph (Abelbeck Software). The peptide CD spectra was analyzed immediately upon dilution and after incubation at 500 μM concentration in buffer P for 24 h. Tryptophan fluorescence was excited at 280 nm and recorded at 340 nm. For calculation of the relative population of folded and unfolded species, the linear-dependence of the fluorescence of both the folded and unfolded latexin states on the urea concentrations was included in the fitting of the experimental data.

### Dye binding assays

Thioflavin-T binding assays were carried out using aliquots of 100 μl drawn from 1 mg/ml protein samples in buffer N after 24 h incubation. These aliquots were diluted into buffer (10 mM sodium phosphate, 150 mM NaCl) containing 65 μM thioflavin-T, and adjusted to a final volume of 1 ml. Fluorescence data were collected after five minutes to ensure that thermal equilibrium had been achieved. Fluorescence emission spectra were recorded using an excitation wavelength fixed at 440 nm. The Th-T stained samples were also analyzed under UV light using a Leica DMBR microscope. Samples were tested for amyloid-specific congo red binding by the spectroscopic band-shift assay as described by Klunk [18]. Briefly, aliquots of 1 mg/ml protein were diluted in reaction solution (5 mM sodium phosphate/150 mM NaCl, pH 7.0) containing 5 μM congo red and absorption spectra were collected together with negative control solutions of dye in absence of protein and of protein samples in the absence of dye, subtracting the absorption of the dye and the scattering contribution from the samples spectra.

### Transmission electron microscopy

Samples containing 1 mg/ml of latexin or 500 μM of VLHLAWVA peptide were incubated as above. A 5 μl aliquot was then placed on carbon-coated copper grids, and allowed to stand for two minutes. The grids were then washed with distilled water and stained with 2% (w/v) uranyl acetate and allowed to stay for two minutes prior to analysis using a Hitachi H-7000 transmission electron microscope operating at an accelerating voltage of 75 kV.

### ATR-FTIR spectroscopy

The VLHLAWVA peptide was incubated in buffer P for 24 h at room temperature and prepared essentially as previously described [43]. Briefly, the microcrystals were lyophilized before analysis to reduce water interference in the infrared spectra and the sample applied on top of the germanium crystal in the attenuated total reflection module of a Bruker Tensor FT-IR spectrometer. The structure of the peptide aggregates was then analyzed. For each spectrum, 20 interferograms were collected and averaged. All processing procedures were carried out so as to optimize the quality of the spectrum in the amide I region, between 1550 cm^-1^and 1700 cm^-1^.

## List of Abbreviations

ATR-FTIR, Attenuated Total Reflectance Fourier Transform Infrared; CD, circular dichroism; EM, electron microscopy; TFA, trifluoroacetic acid; Th-T, thioflavin-T; TNS, 2-p-toluidinylnaphthalene-6-sulfonate.

## Authors' contributions

IP performed most of the experiments and prepared the final data and figures. CB contributed to protein production. FXA and JV contributed to data interpretation and manuscript writing. SV directed the work and prepared the manuscript. All authors read and approved the final manuscript.
